# Upregulation of a Small-World Brain Network Improves Inhibitory Control: An fNIRS Neurofeedback Training Study

**DOI:** 10.3390/brainsci13111516

**Published:** 2023-10-26

**Authors:** Lingwei Zeng, Chunchen Wang, Kewei Sun, Yue Pu, Yuntao Gao, Hui Wang, Xufeng Liu, Zhihong Wen

**Affiliations:** 1 Department of Medical Psychology, Fourth Military Medical University, Xi’an 710032, China; lngwii@fmmu.edu.cn (L.Z.); xlxsunkewei@126.com (K.S.); puyue199901@163.com (Y.P.); gaoyuntao@fmmu.edu.cn (Y.G.); huiwang@fmmu.edu.cn (H.W.); 2 Department of Aerospace Medicine, Fourth Military Medical University, Xi’an 710032, China; ccwang@fmmu.edu.cn

**Keywords:** inhibitory control, small-world network, functional brain network, functional near-infrared spectroscopy, neurofeedback training

## Abstract

The aim of this study was to investigate the inner link between the small-world brain network and inhibitory control. Functional near-infrared spectroscopy (fNIRS) was used to construct a neurofeedback (NF) training system and regulate the frontal small-world brain network. The small-world network downregulation group (DOWN, *n* = 17) and the small-world network upregulation group (UP, *n* = 17) received five days of fNIRS-NF training and performed the color–word Stroop task before and after training. The behavioral and functional brain network topology results of both groups were analyzed by a repeated-measures analysis of variance (ANOVA), which showed that the upregulation training helped to improve inhibitory control. The upregulated small-world brain network exhibits an increase in the brain network regularization, links widely dispersed brain resources, and reduces the lateralization of brain functional networks between hemispheres. This suggests an inherent correlation between small-world functional brain networks and inhibitory control; moreover, dynamic optimization under cost efficiency trade-offs provides a neural basis for inhibitory control. Inhibitory control is not a simple function of a single brain region or connectivity but rather an emergent property of a broader network.

## 1. Introduction

Researchers have theorized that the human brain has significant small-world properties, which are the result of natural selection of the brain under a cost–efficiency balance [1,2,3]. However, there is no consensus on how the small-world network architecture corresponds to better brain function.

Some studies have found that the small-world properties of the brain can predict behavioral or nervous system diseases related to inhibitory control. For example, the core defect of patients with attention deficit hyperactivity disorder (ADHD) is inhibitory control impairment; their brain network topology shows an increase in the local efficiency and a decrease in the global efficiency, and the brain network exhibits changes that are more similar to those of a regular network [4,5,6]. Through the study of the prefrontal cortex of healthy people, subjects with spontaneous behavior showed a larger clustering coefficient, shorter average path length and stronger randomness than those with self-control behavior [7], and self-control behavior reflected inhibitory control [8,9,10]. In the normal population, the functional connectivity brain network has been proven to flexibly adapt to changing cognitive needs [11], and changes in the cognitive state or cognitive capacity might be associated with (potentially rapid) changes in the configuration of brain functional networks [2]. These findings suggest that there is an inner link between the small-world network and inhibitory control.

To investigate this issue, we utilized a functional near-infrared spectroscopy neurofeedback (fNIRS-NF) training method to regulate the human small-world brain network. Compared with traditional electroencephalogram (EEG) and functional magnetic resonance imaging (fMRI) neurofeedback methods, fNIRS has a moderate spatial resolution, temporal resolution, and signal-to-noise ratio. In addition, fNIRS has better usability, is not sensitive to head movement, and is suitable for real-time neurofeedback and clinical application. FNIRS-NF training allows individuals to learn to control and regulate their brain activities [12,13]. Operant learning theory, often used to explain neurofeedback learning, states that the probability of a physiological response is increased when a reinforcing stimulus follows that response. The theory focuses on three main elements: (1) discriminative stimuli, (2) responses, and (3) reinforcers [14]. In addition, the behavioral changes that have been observed to result from the self-manipulation of neural activation and the physiological consequences of neurofeedback may be considered a form of endogenous neural stimulation [15,16], which can be used to clarify the cause-and-effect relationships between functions and brain activity [15,17,18,19].

Researchers have found that neurofeedback training can regulate brain network connections and change brain activity during relevant stimulation [20,21,22] and is considered a potential treatment for disinhibited, antisocial, and violent behavior [23,24,25]. However, there is no consensus on the mechanism by which feedback training alters inhibitory control. Some studies suggest that repeated training enhances the performance of the brain’s ‘muscles’ [8,26], while others suggest that the alteration is due to the changes in the connectivity between brain regions that lead to changes in cognition and behavior [27,28]. However, a recent study showed that no neuron is an island, and functions are an emerging property of the interaction between brain areas [29]. Therefore, global network metrics can help us better understand individual differences and identify the reasons for functional emergence.

Humphries defined a precise measure of ‘small-worldness’ according to the trade-off between high local clustering and short path length [30]. The metrics of a normalized characteristic path length (λ) and clustering coefficient (γ) compared with random networks were introduced, and small-worldness (σ = γ/λ) was derived from these metrics to determine a quantitative, continuous grading of network status.

Some studies have suggested that brain regions that support executive functions seem to be particularly plastic and responsive to environmental changes [31]. The prefrontal cortex (PFC) is the key brain region involved in executive function and plays a central role in goal-directed behaviors [32]. In this study, we hypothesized that the regulation of the PFC small-world network may lead to changes in executive function, especially inhibitory control. Cognitive and behavioral changes can be explained within the framework of the small-world brain network, as it represents the dynamic optimization of the brain under cost–efficiency trade-offs.

## 2. Materials and Methods

### 2.1. Trial Setting and Sample Size

The trial was conducted in Xi’an, China from September 2022 to May 2023. The study was approved by the Ethics Review Committee of the Fourth Military Medical University, and all the subjects carefully read and signed the informed consent form for the cognitive experiments. The experiment adopted a randomized, single-blinded, and mutual control design with different regulating directions according to the study by Yamashita [33]. 

The study consisted of cognitive performance measurements at three time points: before the training (pretest), 1 day after the training (posttest), and at a follow-up assessment 1 week after the end of training (follow-up test).

The sample size was calculated by G*Power version 3.1, where the mixed-design repeated-measures ANOVA model was adapted. We expected a medium effect on the inhibitory control performance based on a previous NF study that reported a medium to large effect on executive functions [34]. The effect size was set at 0.25, α = 0.05, 1 − β = 0.8, and the number of measurements was 3. The required minimum sample size was 28 (14 participants in each group).

### 2.2. Participants

A total of 40 healthy adults were recruited to participate in this experiment. They were all medical college students, excluding patients with brain trauma, mental illness, and recent cognitive experiments. Twenty subjects were randomly assigned to the DOWN group and 20 subjects to the UP group while balancing sex, age, and educational background. All the subjects completed the Edinburgh inventory [35], and all of them were right-handed. The data from 6 subjects were excluded due to incomplete data records. The data from 17 subjects in the DOWN group (10 females and 7 males) and 17 subjects in the UP group (9 females and 8 males) were used in this study. We used the independent samples *t*-test to compare the age and education between the groups, and there was no significant difference, as shown in Table 1.

### 2.3. Brain Imaging Tools and Region of Interest Definition

**Figure 1:**
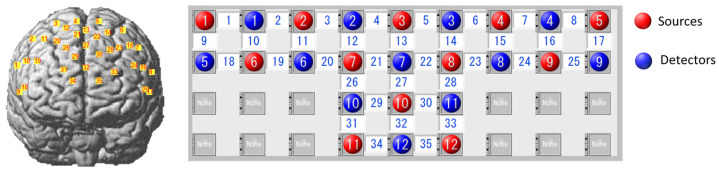
Channel layout and position of light sources and detectors.

In this study, a Shimadzu LABNIRS optical brain functional imaging device was used to monitor the concentration of hemoglobin in the cerebral cortex by a three-wavelength near-infrared semiconductor laser (780 nm, 805 nm, 830 nm). The sampling rate was set to 25.6 Hz. The channel layout mainly covered the frontal lobe, the light source and detector were fixed with a nylon cap, and the distance between the light source and the detector was fixed at 3 cm. Note that there was no short channel employed in our experiment. A total of 12 sources and 12 detectors were arranged for a total of 35 channels, as shown in Figure 1.

The actual coordinates of the channels were obtained by using the Fastrak3d locator, and then the corresponding relationship between the MNI coordinates and the Brodmann area was obtained by probability registration, as shown in Table 2.

### 2.4. Training Procedures and Behavioral Testing

**Figure 2:**
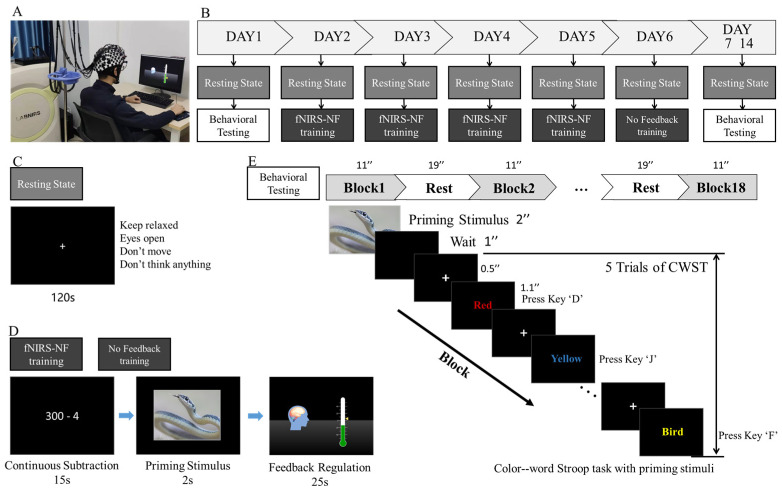
Experimental procedures. (**A**) The fNIRS-NF training scenario. (**B**) The full training and testing schedule. (**C**) The resting state testing. (**D**) A single-block procedure of fNIRS-NF training and no feedback training. (**E**) The behavioral testing before and after fNIRS-NF training. “+” is the fixation.

The subjects sat in front of the computer with their eyes 60–70 cm away from the screen; kept their bodies, especially their heads, free from strenuous activity; and kept themselves relaxed before training, as shown in Figure 2A.

The neurofeedback training program lasted for a total of 8 days, including behavioral tests on Days 1, 7, and 14 and 5 training sessions from Days 2 to 6, of which the first 4 sessions were training with feedback and the last session (Day 6) was training without feedback. The daily training consisted of 18 blocks. At the end of every 6 blocks, there was a rest period for 60 s. The full training schedule is shown in Figure 2B.

Before the daily training session, the subjects maintained a resting state for 120 s, during which they were asked to open their eyes and relax, keep their body and head still, and try not to think or recall anything during the test, as shown in Figure 2C.

The instructions for the training session appeared after the end of the resting state, which required the subjects to repeat three main tasks. For Task 1, subjects were asked to perform continuous subtraction according to the instructions for 15 s to limit their cognitive state as the baseline [36]. In Task 2, a picture stimulus was presented for 2 s. Immediately after the presentation of the picture stimulus, the subjects entered Task 3: neurofeedback regulation lasting for 25 s. The single-block task procedure is shown in Figure 2D.

In the neurofeedback task, a green thermometer indicator bar and a yellow triangular slider were displayed on a black screen, where the slider represents the immediate brain network small-worldness (refresh rate 3 Hz for the calculation method; see Section 2.5: Online calculation of the feedback score), while the thermometer indicator bar represents the cumulative value of the brain network small-worldness in the block (refresh rate, 3 Hz). The subjects were instructed to increase (for the UP group) or decrease (for the DOWN group) the thermometer value as much as possible. Subjects could use any psychological strategy other than breathing adjustment, physical changes, or facial expression movement. Subjects were informed that an additional monetary reward (up to CNY 900) would be paid in proportion to their increased or decreased scores, and they received this money after all experiments were completed.

The color–word Stroop task (CWST, 4 colored Chinese characters) with a priming stimulus [37,38] was used in this study as the behavioral test during the pretest, posttest, and follow-up tests. In the experiment, one block consisted of five consecutive trials. Each priming stimulus was presented for 2 s. Then, a black screen was presented for 1 s, a fixation cross was presented for 0.5 s, and each color–word trial was presented for 1.1 s. The participants rested for 17–21 s at the end of each block before beginning the next, for a total of 18 blocks. All trials and blocks were arranged in a pseudorandom order, and it took approximately 9 min to complete the experiment, as shown in Figure 2E.

The priming stimuli were derived from the International Affective Picture System (IAPS) with high arousal [39], and the number of positive and negative images was balanced to counteract the valence. The reason for using emotional pictures as priming stimuli was that emotion has a particularly strong influence on memory [40], so it can enhance the training effect.

### 2.5. Online Calculation of the Feedback Score

We used the in-house MATLAB R2017a (The MathWorks Inc., Natick, MA, USA), including the brain network computation modules of Gretna v2.0.0 [41], for online processing. This software ran on a connected computer and accessed data files in the NIRS system.

The sampling rate of the fNIRS equipment was 25.6 Hz, and the segment length was 128 points; a time window of 5 s was provided to calculate the feedback score of the brain network. To reduce the physiological noise caused by heartbeat, respiration, and other physiological processes, the recorded fNIRS signals were filtered with a Butterworth band pass filter with cut-off frequencies of 0.01 and 0.2 Hz. First, the oxyhemoglobin (OxyHb) data 5 s before the current time point of all channels were recorded, and the connection strength, *r*, between all channel pairs was calculated by the Pearson correlation method. Because the correlation coefficient did not meet the requirements of a normal distribution, the *r* value was converted into a *Z* score. The conversion calculation was as follows:

(1) $$Z=\frac{ln(1+r)}{2ln(1-r)}$$

As a result, a functional connection matrix *M* with a size of 35 × 35 was constructed, with the channel as the node and the *Z* value as the edge. Then, sparsity threshold segmentation was adopted, for example, maintaining 30% (sparsity = 0.3) of the connections with larger Z values. Finally, a binary, undirected network was constructed and used to calculate the small-worldness, *σ_i_*, of the brain network at the *i*th time point. The small-worldness was calculated by the method of Humphries [30] using the Gretna toolbox version 2.0.0. The parameter configurations are shown in Table 3.

The same method was used to calculate the sequence of the small-worldness at baseline (continuous subtraction period); the mean value of the sequence was *σ_base_*, the standard deviation was *SD*, and the feedback score was corrected by the following formulas [22]:

(2) $${Score}_{i}=\frac{50\left(\sigma_{i}+3SD-\sigma_{base}\right)}{3SD}\left(0\leq {Score}_{i}\leq 100\right)$$

Here, *σ_i_* is the small-worldness of the *i*th trial, and *σ_base_* and *SD* are the mean and standard deviation of the small-worldness values at baseline, respectively. The feedback score provided the participant with the following information: the baseline performance corresponded to a score of 50, and a monetary reward was provided if the feedback score of the current trial exceeded 50 (i.e., the frontal small-worldness was higher than the baseline). The scores that dropped below 0 or exceeded 100 were kept at 0 or 100, respectively. The online signal processing and all visual presentations in the experimental protocol were performed using MATLAB software.

### 2.6. Offline Data Analyses

The feedback scores of the DOWN group and UP group were analyzed by a mixed-design repeated-measures ANOVA using IBM SPSS Statistics 23 (The IBM Corp., Chicago, IL, USA). To investigate whether the UP group obtained higher feedback scores during training compared to the DOWN group, we performed independent sample *t*-tests for each training period separately.

To evaluate the cognitive performance, we calculated the score changes in the cognitive performance (the posttest score minus the pretest score; the follow-up test score minus the pretest score). To investigate whether the UP group showed an improved cognitive performance compared to the DOWN group, we analyzed the all-change scores for the cognitive performance using a mixed-design repeated-measures ANOVA in SPSS 23. The scores of the cognitive performance scores analyzed in this study included the accuracy, reaction time, and the Stroop effect.

For the offline NIRS data, all analyses were conducted using Homer 2 [42] and MATLAB 2017a. We performed the following preprocessing steps: (1) Converted intensity (raw data) to optical density (OD). (2) Identified motion artifacts based on amplitude and standard deviation thresholds. (3) Performed a cubic spline correction of the motion artifacts identified in step 2. (4) Applied a 5-order Butterworth band pass filter (cut-off frequencies: 0.01–0.2) to the data to further reduce noise [43]. (5) Converted the OD data to concentrations. As young adult participants were included in our experiment, we chose [6.0 6.0 6.0] as the differential pathlength factor (DPF), which was suggested in Chiarelli’s study [44]. (6) The normalized characteristic path length (λ) and the normalized clustering coefficient (γ) using a method similar to that described in Section 2.5 (Online calculation of the feedback score) for small-worldness (σ). Unlike the online calculation, we used 25 s as a time window, and did not use the baseline correction based on the continuous subtraction periods.

To investigate whether the UP group showed improved brain activity during training compared to the DOWN group, we performed independent sample *t*-tests for each training period separately. In addition, we investigated the association between the changes in inhibition (Stroop effect) and brain activities (small-worldness) and performed a Pearson correlation analysis.

To investigate whether the UP group displayed improved brain activity during training compared to the DOWN group, we separately performed independent sample *t*-tests for each training period. In addition, we investigated the association between the changes in inhibition (Stroop effect, posttest minus pretest) and the brain activities (small-worldness, 5th training without feedback) and performed a Pearson correlation analysis.

In order to investigate the change in the degree distribution in response to the upregulation training compared with that in response to the downregulation training, we averaged the degree distribution of all trials for each subject, and used the independent sample *t*-tests for each degree value. Furthermore, to investigate the impact of the upregulation training on the topology of brain networks, we calculated the brain network topology of the UP group and the DOWN group separately and performed an independent sample *t*-test for each edge. A Pearson correlation analysis was used to investigate the association between the degree values and changes in edge for all participants.

In these analyses, *p* < 0.05 was considered significant for multiple comparison methods using false discovery rate (FDR) correction methods [45].

## 3. Results

### 3.1. Change in Feedback Score

**Figure 3:**
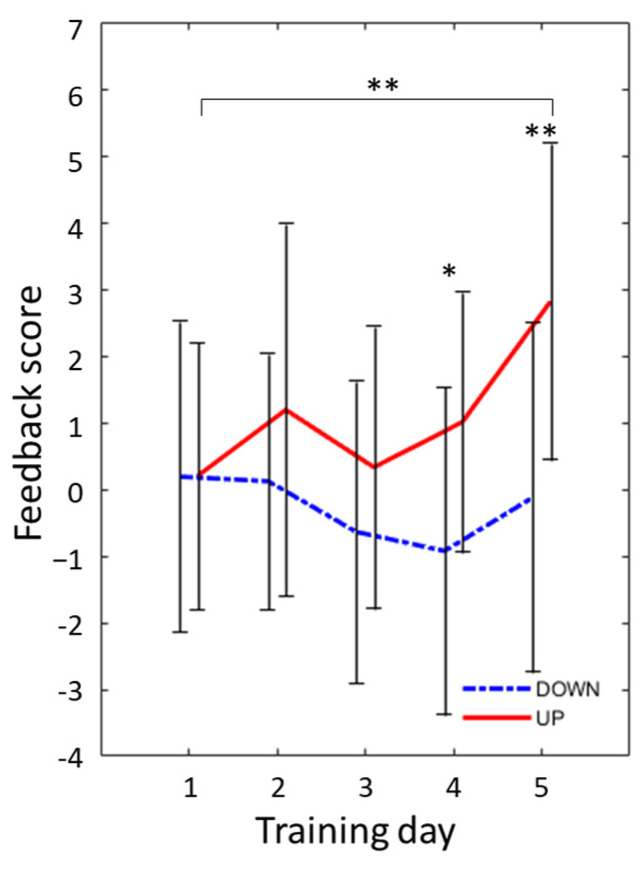
The learning curve of the feedback score. The UP group was significantly higher than the downregulation group after the 4th training day. The red solid line represents the upregulation group, and the blue dotted line represents the downregulation group. Data show the mean ± SD (error bars). A paired *t*-test was used for within-group analyses, and an independent sample *t*-test was used for between-group analyses; * *p* < 0.05, ** *p* < 0.01.

Although the main effect and interaction were not significant, the scores in the DOWN group showed a downward trend on the second, third, and fourth training days, while those in the UP group showed an upward trend on the second, fourth, and fifth training days. The feedback scores of the DOWN group and the UP group were significantly different after the fourth day. On the fourth training day, the score of the DOWN group was significantly lower than that of the UP group (mean ± SD = −0.925 ± 2.459 vs. 1.006 ± 1.948, respectively; *p* = 0.016). On the fifth training day, the score of the DOWN group was also lower than that of the UP group (mean ± SD = −0.123 ± 2.616 vs. 2.813 ± 2.368, *p* = 0.002). In the UP group, the score on the fifth training day was significantly higher than that on the first training day (mean ± SD = 2.813 ± 2.368 vs. 0.200 ± 2.001, *p* = 0.007), as shown in Figure 3.

### 3.2. Change in Cognitive Performance

We applied a mixed-design repeated-measures ANOVA to the accuracy and Stroop effect and examined whether the cognitive performance was enhanced after training. As a result, the interaction effect between the group and day was significant for the Stroop effect (F (2, 64) = 8.113, *p* = 0.001) but not for the accuracy (F (2, 64) = 1.998, *p* = 0.158). These significant interactions suggest that the changes in the Stroop effect from the pretest to the posttest were different between the groups. We also applied a mixed-design repeated-measures ANOVA to the reaction time in the neutral condition. The results revealed that there was a significant main effect on the day (F (2, 64) = 13.620, *p* < 0.001), suggesting a practice effect of the reaction time caused by repeated tests.

**Figure 4:**
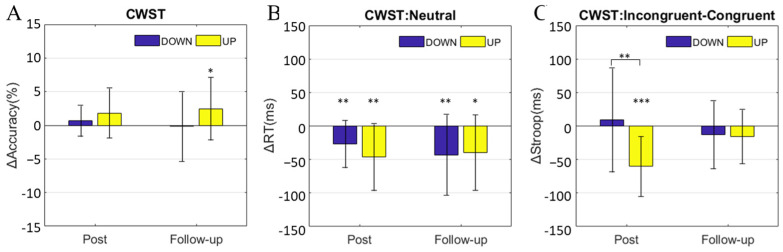
Behavioral test results. (**A**) Change in CWST accuracy, posttest minus pretest; (**B**) change in reaction time in the neutral condition of the CWST, posttest minus pretest; (**C**) change in the Stroop effect of the CWST, posttest minus pretest, posttest minus pretest. All data show the mean (bars) ± SD (error bars). A paired *t*-test was used for within-group analyses, and an independent sample *t*-test was used for between-group analyses; * *p* < 0.05, ** *p* < 0.01, *** *p* < 0.001.

The change in the accuracy (ΔACC) was calculated by subtracting the pretest values from the posttest values, and the ΔACC of the CWST was analyzed by a zero test within a group and an independent sample T test between groups. For the CWST during the follow-up test, the accuracy of the UP group significantly increased (mean ± SD = 2.48 ± 4.69%, *p* = 0.044), but there was no significant difference between groups. The results are shown in Figure 4A.

The change in the RT (ΔRT) was obtained by subtracting the pretest values from the posttest values. There was no significant difference in the CWST in the neutral condition between the groups, but all the RTs of the posttest and follow-up test were significantly decreased compared to those of the pretest (zero-test, posttest of DOWN group: *p* = 0.006; posttest of UP group: *p* = 0.002; follow-up test of DOWN group: *p* = 0.010; follow-up test of UP group: *p* = 0.010), as shown in Figure 4B.

The Stroop effect was calculated by incongruent RT minus congruent RT, which represented the inhibitory control performance of the subject. The change in the Stroop effect (ΔStroop) was obtained by subtracting the pretest values from the posttest values, and the ΔStroop of the CWST was analyzed by a zero test within groups and an independent sample T test between groups. During the posttest, the ΔStroop of the UP group was significantly lower than 0 (mean ± SD = −60 ± 45 ms, *p* < 0.001), and the ΔStroop of the UP group was significantly lower than that of the DOWN group (mean ± SD = −60 ± 45 ms vs. 9 ± 77 ms, *p* = 0.003), as shown in Figure 4C.

### 3.3. Change in Small-World Properties

The fNIRS OxyHb time series was segmented at equal intervals of 25 s without overlapping, and the topological properties of the regulating state and resting state were calculated as described in Section 2.6 (Offline Data Analyses). We analyzed the small-world network under different sparsity thresholds ranging from 0.1 to 0.3 over five training days.

**Figure 5:**
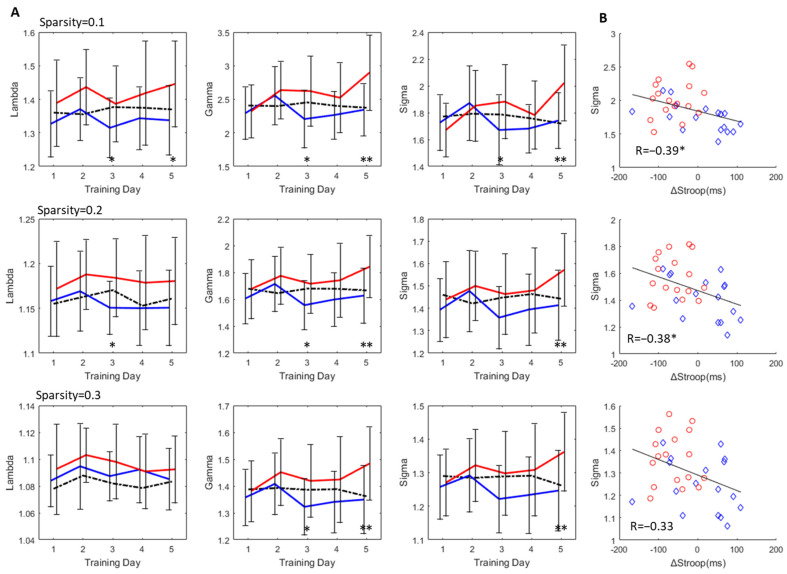
Offline analysis of topological properties of the brain network. (**A**) The changes in the lambda, gamma, and small-worldness (sigma) at different sparsity and different training days, in which the black dotted line represents the average value in the resting state, the blue solid line represents the regulating value of the DOWN group, and the red solid line represents the regulating value of the UP group. (**B**) The Pearson correlation between sigma on the last training day and ΔStroop of CWST (posttest minus pretest), where sparsities of 0.1, 0.2 and 0.3 were calculated. The horizontal axis represents ΔStroop, while the vertical axis represents sigma. The red circles represent the DOWN group and the blue diamonds represent the UP group. An independent sample T test was used in this study. Data show the mean ± SD (error bars). A paired *t*-test was used for within-group analyses, and an independent sample *t*-test was used for between-group analyses; * *p* < 0.05, ** *p* < 0.01.

There were no significant differences between the groups or within the groups during the training in the resting state, but there was a significant difference in the regulating state in the given sparsity of the topological properties. For instance, the sigma of the UP group was significantly larger than that of the DOWN group on the fifth training day (sparsity = 0.10: *p* = 0.002, t = −3.304; sparsity = 0.2: *p* = 0.007, t = −2.892; sparsity = 0.3: *p* = 0.008, t = −2.829); the gamma of the UP group was significantly larger than that of the DOWN group on the fifth training day (sparsity = 0.10: *p* = 0.002, t = −3.357; sparsity = 0.2: *p* = 0.007, t = −2.875; sparsity = 0.3: *p* = 0.005, t = −2.982). Moreover, the DOWN group had only a small deviation from the resting state, while the UP group exhibited a greater deviation, as shown in Figure 5A.

We then analyzed the relationship between the small-world networks and cognitive enhancement and used the small-worldness of the fifth training session (non-feedback) to perform a Pearson correlation analysis with the ΔStroop (posttest minus pretest). The results showed that the ΔStroop was significantly negatively correlated with small-worldness when the sparsity was lower than 0.3 (sparsity = 0.10: *p* = 0.021; sparsity = 0.2: *p* = 0.029, respectively), as shown in Figure 5B.

The changing pattern in the offline calculated brain network small-worldness is consistent with the feedback score of the online calculation, as shown in Figure 3. According to the changes in lambda, gamma, and sigma, we found that the UP group significantly increased the clustering coefficient but slightly decreased the characteristic path length; that is, gamma contributed more to brain network changes than lambda.

### 3.4. Degree Distribution and Edge Analysis

**Figure 6:**
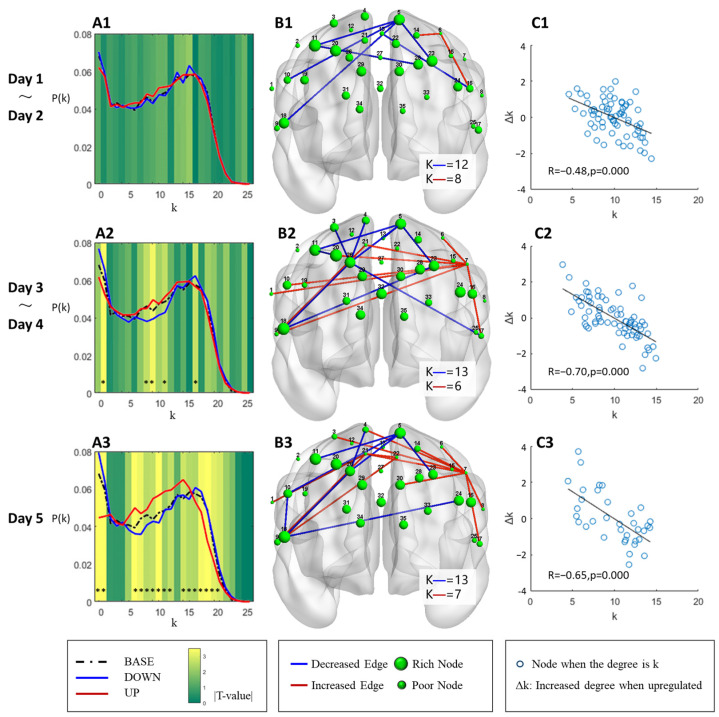
Degree distribution and edge reallocation. (**A1**–**A3**) The degree distribution curves of the DOWN group (blue solid line) and UP group (red solid line) in different training periods. The black dotted line represents the average degree distribution before training, and different background colors represent the absolute value of the T value of the independent sample T test between groups, * *p* < 0.05. (**B1**–**B3**) The increased edge (red color edge) and the decreased edge (blue color edge) of the UP group compared with the DOWN group in different training periods by the independent sample T test where *p* < 0.01 was selected for drawing. The green node is the position of the channel, and its size represents the degree of the node. ‘K—’ indicates the average degree of the nodes connected to the red line and the blue line, respectively. (**C1**–**C3**) The correlation between the degree of all nodes and their edge changes, where the horizontal coordinate (k) is the node degree, the vertical coordinate (Δk) is the increment in the node degree when upregulating the small-world network, R is the Pearson correlation coefficient, and *p* is the significance.

We selected a sparsity of 0.3 for the edge analysis. The degree distribution of the DOWN group and the UP group gradually showed differences across the five training days. In the fifth non-feedback training, the probability of nodes in the DOWN group was significantly greater than that in the UP group at degree values from 0 to 1 (mean ± SD = 0.072 ± 0.026 vs. 0.045 ± 0.018, respectively; *p* = 0.001). The probability of nodes in the DOWN group was significantly less than that in the UP group (mean ± SD = 0.045 ± 0.009 vs. 0.055 ± 0.008, respectively; *p* = 0.001) when the degree value was between 6 and 15. When the degree value was between 16 and 20, the probability of nodes in the DOWN group was significantly greater than that in the UP group (mean ± SD = 0.051 ± 0.008 vs. 0.043 ± 0.007, respectively; *p* = 0.003), as shown in Figure 6(A3). The degree distribution of the DOWN group had two peaks (degree = 0, 16), whereas the UP group showed a bell distribution on the last training day, which is close to the regular network, as shown in Figure 6(A3).

In terms of the edge changes, we defined the UP group minus the DOWN group as the effect of the upregulation of the small-world brain network, which reallocated the edges of some high-degree nodes (rich node, larger green nodes in Figure 6(B1–B3)) to low-degree nodes (poor node, smaller green nodes). Taking the last training day as an example, the average node degree of the blue edges (decreased edges) was 13, while the average node degree of the red edges (increased edges) was 7, as shown in Figure 6(B3), indicating that low-degree nodes tended to increase connections, while high-degree nodes tended to decrease connections when upregulating the small-world network. The scatter plot of the node degree and its alteration are shown in Figure 6(C1–C3). The increased edges of nodes were strongly negatively correlated with the degree of the nodes.

### 3.5. Inference on the Evolution of the Brain Network

**Figure 7:**
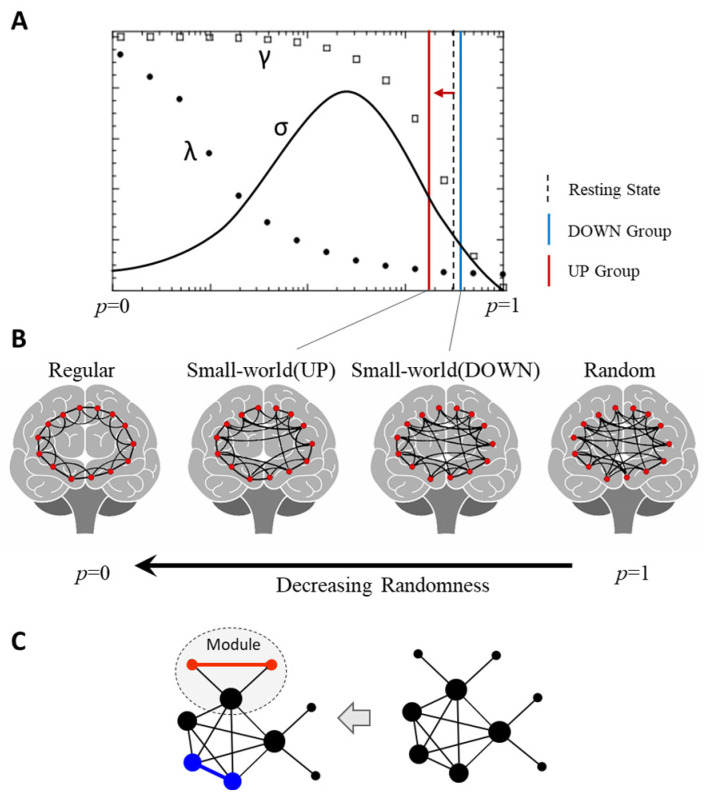
Directional regulation changes the randomness of the brain network. (**A**) The position of the DOWN group (blue solid line) and the UP group (red solid line) in the randomly rewired graph (nonnumerical result). (**B**) The UP group changed to be more similar to the lower randomness of a regular network compared with the DOWN group. (**C**) A possible method of brain network regulation (from DOWN to UP): the UP group disconnected some high-degree nodes (blue edges) and reconnected them to low-degree nodes to form a triangular loop (red edges).

The increase in gamma and lambda indicates a decrease in the random rewiring probability based on the small-world model. As shown in the results in Section 3.3, upregulation resulted in a significant increase in the clustering coefficient and a slight increase in the path length, so we inferred and roughly located the position of both groups in the family of randomly rewired graphs, as shown in Figure 7A. Although this is not a numerical result, we can conclude that the UP group changed the network to a lattice-like regular network and moved away from the random network compared with the DOWN group, as shown in Figure 7B. These results also allowed us to simulate the evolution of brain networks. A possible method of brain network regulation is shown in Figure 7C, where high-degree nodes are disconnected and low-degree nodes are connected to increase the probability of triangular loops.

## 4. Discussion

In this study, the fNIRS neurofeedback training method was used to regulate the functional brain network of the human frontal cortex. A randomized, single-blinded, and mutual control design with opposite regulating directions assigned to different groups was adopted in the present experiment to eliminate general effects such as the placebo effect, which referred to other neurofeedback training studies [33,46,47].

In this experiment, a reinforcer could be the real-time feedback of the monetary reward in the form of an increase in the bars of a thermometer in proportion to the small-worldness of the brain network relative to a given reference activity. In the cognitive test, learning mechanisms extract useful information from experience, while memory carries the acquired information forward in time in a computational form that can be retrieved and used by the subject [14]. The experimental results showed that this method has a training effect on the brain network topology and can accordingly improve inhibitory control.

Small-worldness feedback of the brain network has the advantage of being independent of the selection of the brain region and channel location and is the global perspective of the measurement area. The scaffolding theory of aging and cognition suggests that cognitive training in elderly individuals may improve function by increasing the neural activity in compensatory regions [26,48]. Therefore, brain functional regions may have compensatory changes in life, and this new approach may have the same training effect on compensatory areas and even assist in establishing new compensatory brain regions and functions for some patients with mild cognitive impairment. The findings are expected to be applicable to people with mental disorders related to inhibitory control impairment.

Although oxyhemoglobin concentration was used in this study, it does not mean that deoxyhemoglobin (DeoxyHb) concentration is not available. Our additional analysis showed that the change in the DeoxyHb concentration can partly predict the change in inhibitory control. This is in line with another study in which the stability and reliability of DeoxyHb cannot be compared with OxyHb [49].

### 4.1. Reducing the Randomness of the Brain Network Accompanied by Improved Inhibitory Control

Neurofeedback training based on the small-world network model can significantly change the topology of the brain network. The scores of the UP group increased significantly during the training sessions, and the regulation difficulty in different directions was inconsistent. First, the UP group seemed to obtain higher scores easier, while the DOWN group had more difficulty; in addition, the score of the UP group on the fifth training day was significantly higher than that on the first training day, which suggested that upregulation seems more likely to be successful. This result was consistent with other studies where regulation was primed by stimuli [50,51]. A previous functional connectivity study showed that when a human is passively watching highly arousing visual stimuli, the brain’s small-world properties seem to be decreased since the cluster coefficient and global efficiency are reduced [52], and the UP group recovered from the reduced small-worldness more easily, while the DOWN group demonstrated more difficulty in maintaining or further reducing the small-worldness.

The analysis of the self-reported regulating strategies of the subjects showed that the UP group tended to adopt a strategy that made them happy and excited, while the DOWN group tended to calm themselves down. We inferred that upregulation is a method of brain optimization and improving cognitive performance for subsequent activities, while downregulation shows a tendency to inhibit brain changes and leads to exhaustion.

A previous study reported that subjects with spontaneous behavior showed a larger clustering coefficient, shorter characteristic path length, and stronger randomness than those with self-control behavior [7], namely, stronger inhibitory control requires higher network regularity, which is consistent with the results of this study. We should note that the regularized network is a state with more clustering and is less costly [53,54] in cases of reduced cognitive demand, which is a manifestation of cost savings. Regularized changes in healthy individuals are more likely to accumulate cognitive resources for preparing for subsequent tasks. That is, the small-world network is a resource optimized for inhibitory control.

We can improve cognitive performance by changing the topology of the brain network, but this does not mean that any form of change is beneficial; it must be limited by the cost and architecture of the real brain material basis. The correlation analysis between behavior and brain networks indicates that the improvement of inhibitory control indicates its relationship with the small-world network. However, small-world networks are unlikely to support all cognitive performance but are specific because the dynamic changes in the brain network are driven by task requirements [2,11].

### 4.2. Changes in the Small-World Brain Network and Causal Inference

Studies are generally based on the assumption that anatomical networks have significant small-world properties, which is the result of optimizing an economic trade-off between the cost and the behavioral value of the network function [2,11]. However, the functional brain network is different from the anatomical network. The connections in the functional brain network are obtained by statistical inference and binary segmentation, which highlights the different features of the functional brain network, such as a stronger dynamic configuration ability and more plasticity, but it may introduce more noise and increase the randomness of the network. In fact, when we reduced the sparsity, the small-worldness increased, which implied that removing some ambiguous connections may help further accurately estimate the brain network. When the sparsity was 0.1, the small-worldness was approximately 2.0, which is consistent with the results of other functional brain network studies with similar node numbers [2,55,56]. These results indicated that the functional connectivity network of the frontal lobe has a significant small-world property as well as an anatomical network.

An important contribution of this study is the depiction of the regulation process of small-world networks. First, small-world network regulation is a robust process that has many of the same features under different sparsities as other studies [7,52,57]. Second, it is a gradual process, and the changing patterns of the brain network at different training phases are consistent. Third, the position of the brain network in the family of randomly rewired graphs can be roughly estimated by analyzing the change trend of lambda and gamma, which was consistent with the result of the study by Cao [4].

Focusing on the changing pattern in the upregulation, because the number of edges is constant according to the small-world model, poor nodes are connected, while rich nodes are disconnected. The role of feedback is to enable the brain to better identify which edges are not important and thus can be disconnected, and which nodes are isolated and need to be connected first (this may be achieved in a trial-and-error manner) [58]. Even if not driven by a cognitive task, the self-reshaping of the brain network guided by this simple principle miraculously supports cognitive performance. This provides strong support for the causal inference between small-worldness and inhibitory control.

Another important feature is that, in addition to eliminating the imbalance of degree distribution, upregulation also reduces the spatial asymmetry between hemispheres. In terms of increasing and decreasing edges, the increased edges in the UP group were mainly concentrated in the left frontal eye fields, while the decreased edges were mainly concentrated in the right dorsolateral prefrontal cortex and the left premotor and supplementary motor cortex. The altered edges showed an increase in density but maintained a good regional consistency during the different training periods, as shown in Figure 6B.

In general, upregulation training reconfigures a small-world network with more functional specificity and a modular structure, which is characterized by linking widely dispersed brain resources and supporting efficient processing of specific tasks. The brain network of the upregulation changed to be more consistent with a lattice-like regular network, but it was still efficient and could save more cognitive resources for the subsequent inhibitory control tasks.

Previous studies have shown that small-world topology supports the emergence of complex neurodynamics [59] characterized by the coexistence of functional separation and integration. Other studies have shown that small-world networks contribute to the synchronization and dissemination of information [60,61], enhance computing power [62], and provide many other functional benefits. This study suggests that the small-world brain network is a dynamically callable resource, and the brain can perform better inhibitory control by upregulating the small-worldness. Small-world networks are not only the result of brain optimization trade-offs between cost and efficiency [63] but are also potential targets for regulating the brain efficiency.

### 4.3. Limitations

The analysis of the resting state data revealed that the upregulation training led to an upward trend in the clustering coefficient and small-worldness, which was similar to the results of the regulating state. We did not show them in the results section because their stability and effect size could not be compared with the latter. The gap between the resting state and regulating state may be due to insufficient training times and efficiency. The training in this study was always primed with stimulation. It was stimulus-dependent while strengthening the brain’s self-regulation. To reduce the stimulus dependence, further study should focus on improving the training procedures and increasing the training duration.

In this study, healthy young adults were selected as the subjects, and the influence of elderly individuals and juveniles on the regulation of the small-world network was not fully investigated. We look forward to further study in the future. In addition, we investigated the impact of sex and found that sex has little influence on the training effect; however, the accuracy of the female participants in the DOWN group was significantly higher than that of the male participants, which may be because the women became more careful than the men when they felt that the self-regulation training was difficult.

In addition, we applied the post hoc power analysis in ANOVAs rather than other T test statistics (such as the analysis of the edges in Section 3.4 and of the degree distribution), which is a preliminary work to illustrate the change pattern of the brain network, and we expect to improve it in a subsequent study.

## 5. Conclusions

The functional human brain network is a small-world network, and fNIRS neurofeedback training based on the small-world network model enables individuals to regulate the brain network topology, although upregulation seems to be better in almost all aspects. Upregulation training helps to improve the inhibitory control accompanied by increasing small-worldness and decreasing randomness, and the brain network is improved to be more regular that links widely dispersed brain resources, not only among nodes but also between hemispheres. These results indicate that inhibitory control is not a simple function of a single brain region or connectivity but rather an emergent property of a broader network. The repeated upregulation training of the small-world network may be beneficial to long-lasting inhibitory control performance.

## Figures and Tables

**Table 1:** Demographic information for participants in each group.

	Number of Participants	Age (SD) in Years	Education Years (SD)
DOWN	17 (10 female)	21.53 (2.00)	15.24 (1.78)
UP	17 (9 females)	20.82 (1.55)	14.36 (1.56)
*p*-Value	--	0.26	0.13

**Table 2:** Correspondence between the Brodmann area and channels.

Brodmann Area	Right Hemisphere	Left Hemisphere
Dorsolateral prefrontal cortex	1, 10, 18, 19, 29	16, 24, 25, 28, 30
Premotor and supplementary motor cortex	2, 3, 4, 12	5, 6, 13
Frontal eye fields	11, 20, 21, 26	7, 14, 15, 22, 23, 27
Pars triangularis Broca’s area	9	8, 17
Frontopolar area	31, 34	32, 33, 35

**Table 3:** Parameter configuration in Gretna.

Parameter	Value
Type of matrix sign	Absolute
Method of thresholding	Sparsity
Threshold sequence	0.3
Type of network	Binary
Number of random networks	30
Algorithm to estimate clustering coefficient	1

## Data Availability

The data presented in this study are available on request from the corresponding author.
